# Novel SWIPT Schemes for 5G Wireless Networks

**DOI:** 10.3390/s19051169

**Published:** 2019-03-07

**Authors:** Akashkumar Rajaram, Rabia Khan, Selvakumar Tharranetharan, Dushantha Nalin K. Jayakody, Rui Dinis, Stefan Panic

**Affiliations:** 1 School of Computer Science and Robotics, National Research Tomsk Polytechnic University, 634050 Tomsk, Russia; razharam@tpu.ru (A.R.); khanrabia@tpu.ru (R.K.); stefanpnc@tpu.ru (S.P.); 2 Faculty of Science and Technology, New University of Lisbon, Portugal, and Instituto de Telecomunicações, 1049-001 Lisbon, Portugal; 3 School of Engineering, Sri Lanka Technological Campus, 10500 Padukka City, Sri Lanka; tharranetharans@sltc.edu.lk

**Keywords:** SWIPT, WPT, cooperative communications, M-NOMA, channel estimation, receiver design

## Abstract

In this paper, we present a few novel simultaneous wireless information and power transfer (SWIPT) schemes that can be effectively used in various 5G wireless network implementations. First, we study the possibility of integrating distributed energy beamforming with the data rate fairness beamforming in a cooperative communication system with multiple cooperative relays and multiple destination users communicating simultaneously. We show that the system exploits significant performance gain using such a joint energy and data rate fairness beamforming scheme. Further, we propose an enhanced version of the SWIPT scheme, the energy-efficient modulation-based non-orthogonal multiple access (M-NOMA) SWIPT scheme, and observe its system efficiency in terms of more harvested energy. Finally, we consider an energy-harvesting SWIPT scheme where the channel response is estimated using the energy-harvesting signal as pilots superimposed on the information signal. For such a scheme, we compute the optimum transmit power ratio between the pilot and information signals under varying SNR conditions and improve the accuracy of the decoding process at the reception.

## 1. Introduction

In the rapidly-evolving 5G wireless communication systems, the network size is expected to increase substantially, namely due to machine-to-machine (M2M) communications and sensor networks [1,2]. Moreover, the user requirements are also expected to be much higher due to a variety of 5G applications. This burdens existing power resources, and the traditional power supply should be complemented by alternative sources of power, namely for sensor devices employed in smart city and indoor Internet of Things (IoT) applications [3]. Those alternative power resources provide flexibility to sensor devices and are particularly useful where a fixed power supply cannot be employed. The alternative power resources include radio frequency (RF) energy and renewable energy resources like solar energy, etc. For several applications where renewable sources are not a viable alternative, like in many indoor applications, RF energy harvesting is a particularly interesting alternative. For RF energy-harvesting schemes, there are in fact two main techniques used for RF energy harvesting: wireless power transfer (WPT) and simultaneous wireless information and power transfer (SWIPT) [4,5,6,7].

The WPT is a technique to transmit power using the radio frequency waves from a dedicated energy source such as a base station to energy-constrained nodes such as mobile users [4,5,6]. The WPT can be performed by a base station, which already exists in the infrastructure and has enough power supply, or by power beacons (PBs), which are specifically deployed in the system as dedicated power-supplying nodes [8,9]. The PBs have gained more attention since they provide better energy efficiency due to their close proximity to end users. Moreover, they are easy to deploy, as they do not require backhaul connection.

SWIPT is a wireless communication technique through which it is possible to receive information and harvest energy from a received signal, and the harvested energy can be utilized for relaying of information or processing purposes [7]. It is helpful for saving energy of the system as a whole through reclamation of energy. WPT and SWIPT have recently gained much attention in both academia and industry, as they open new prospects in wireless communication systems.

On the other hand, cooperative communication using the mobile users in a network can be used to improve the coverage and capacity of traditional wireless communication systems [10]. Moreover, the cooperative beamforming technique provides geographically-distributed cooperative mobile users a single antenna to act cooperatively as an antenna array and beamforming to enhance the performance [11]. However, this system consumes energy from mobile users to enable the cooperation. Thus, mobile users will not participate in cooperation to save their battery life. The WPT is a potential solution to provide the required energy for the cooperation of mobile users [12]. This solution will motivate the mobile users to participate in cooperation and help to improve the system performance.

Motivated by this, we consider a cooperative communication system with multiple cooperative relay users (CRUs) transmitting different information to multiple destination users (DUs) simultaneously. Examples of such a system include the second hop of CRU-assisted cellular communication, where the base station cannot communicate with the DUs directly due to shadowing or the geographical location of DUs. Another example is the second hop of a multiple peer-to-peer communication system via a common set of CRUs, where the sources cannot communicate with their corresponding DUs directly [13,14]. In contrast to the traditional systems, the CRUs harvest energy from dedicated PBs deployed in the system and then use that energy to transmit the information to DUs. In WPT, the efficiency of energy harvesting can be improved by energy beamforming. Therefore, we perform distributed beamforming to maximize the total energy effectively delivered to the CRUs. Then, for such systems, providing fair service to all DUs is important. Therefore, we propose distributed cooperative beamforming to provide data rate fairness to all the DUs. Finally, we propose joint energy and data rate fairness beamforming to improve the overall system performance.

Together with the energy constraints, the design of wireless communication systems is also conditioned by spectral limitations. There is a number of newly-discovered technologies, which are claiming to provide better utilization of the spectrum and energy in terms of fulfilling the forthcoming high demand for the new connected world. Non-orthogonal multiple access (NOMA) is one of the aspirants of the forthcoming 5G technology for better and efficient utilization of the radio spectrum. Early generations including 2G, 3G, and 4G allow access to the radio spectrum orthogonally, via the orthogonal multiple access (OMA) scheme. OMA divides the entire available spectrum in terms of its frequency to multiple users. An user far from the transmitter, possibly in an adjacent cell, can utilize the spectrum allocated to a single user. The least reutilization of spectral resources easily exhausts the spectrum. In contrast, NOMA allows spectrum access either without or with minimum division of the radio spectrum. Therefore, there is no problem in terms of spectrum utilization since it is not restricted to one or a couple of users. Along with better spectral efficiency, NOMA provides fast, reliable, and energy-efficient communication services.

Enabling SWIPT with NOMA in the wireless network system provides an efficient use of the energy and radio spectrum. Many authors put these schemes together and obtained amazing and substantial results. Cognitive radio (CR) networks are a new type of network that use innovative technologies to benefit the system. Many authors put together such innovative beneficial technologies to provide better systems. For a battery-driven power-limited massive population, the authors of [15] proposed SWIPT-enabled MISO NOMA CR networks. For improved secrecy, the proposed technique provided security when combined with artificial noise-aided cooperative jamming. In [16], the authors studied SWIPT-enabled MIMO NOMA cognitive networks for maximizing the harvested energy by the secondary user (relay) and minimizing the transmission power on cognitive BS. In [17], the authors investigated SWIPT cooperative NOMA multi-cell beamforming for physical layer security (PLS) through artificial noise (AN) transceiver optimization. To guarantee quality of service (QoS), the authors formulated the optimal spectral efficiency problem. In the downlink NOMA (DL NOMA) system with SWIPT, the authors of [18] studied the PLS with the optimum power splitting and power allocation problem for maximizing the secrecy sum rate. For maximizing the sum secrecy rate, the authors of [19] studied DL SWIPT-enabled NOMA satisfying the QoS demands of all users. The authors of [20] proposed the MIMO and SISO SWIPT NOMA scheme with joint optimization of power splitting (PS) and beamforming. The latter maximizes the data rate of strong users and the QoS of weak users, where strong users harvest energy and transmit the signal to weak users. In [21], authors proposed SWIPT-enabled cooperative NOMA combined with beamforming. In this paper, the strong user harvests energy and transmits power in full duplex mode. The authors of [15,22], targeted MISO NOMA SWIPT security with the aid of artificial noise beamforming. In one of the sections, we demonstrate the beamforming technique for WPT.

Finally, we discuss the possibility of using the SWIPT protocol for energy harvesting (EH) along with carrier frequency offset (CFO) estimation and channel estimation on a single-carrier frequency-division multiple access (SC-FDMA) signal. SC-FDMA is sensitive to CFO [23]. CFO occurs mainly due to the frequency mismatch between the oscillators at the transmitter and at the receiver [24,25]. Doppler shift was also one of the other reason for CFO [26]. CFO creates frequency-related errors at the receiver, which directly affects the performance of signal detection [27,28]. There were many CFO estimation techniques proposed in [29,30,31]. In [29], the maximum likelihood-based frequency offset estimation technique was proposed, which is suitable for CFO that is within the acquisition range, i.e., ±1/(2T), where *T* is the time duration of the symbol. For CFO within a higher acquisition range, a new technique based on two separate pilot signals was proposed in [30], and further based on this technique, an algorithm called the best linear unbiased estimator (black) was proposed in [31].

At the receiver, we use iterative block decision feedback equalization (IB-DFE) to perform frequency domain equalization, which performs better than non-iterative methods [32,33]. With the help of IB-DFE, the channel estimation is performed by using superimposed pilot symbols, as in [34]. Alternatively, the channel estimation is performed by using multiplexed pilot symbols [35,36]. In block transmission techniques, due to the possibility of very long channel impulse response, the channel estimates are not reliable with multiplexed pilot symbols. Thus, superimposed pilots offer more accurate and reliable channel estimation. On the contrary, due to increased pilot symbols, the superimposed pilot signal uses excess power resources as compared to the multiplexed pilot signal. Thereby, this is a suitable candidate for SWIPT, where we can use the excessive power used by the pilot signal for EH [37]. The power allocation and optimization comprise one of the important resource management features in SWIPT, which improves spectral efficiency and overall system performance, as mentioned in recent articles [38,39,40,41]. In this case study, we study the optimum power ratio between pilot and information symbols.

The study is organized as follows: In Section 2, we explain the summary of the case studies and the motivation for the case study. In Section 3, we will observe the case study of WPT-enabled data rate fairness beamforming. In Section 4, we will observe the case study of built-in energy-efficient modulation-based NOMA. In Section 5, we will provide the case study of SWIPT receiver design with joint CFO and channel estimation, Finally, concluding remarks are provided in Section 6.

## 2. Motivation for an Energy-Efficient 5G Network

In this instructional paper, we consider three different case studies. The techniques that are used in the case studies are beamforming, NOMA, and SC-FDMA. SC-FDMA is an energy-efficient signal transmission technique for uplink communication in 5G [42,43]. The first case study considers WPT-enabled cooperative communication systems, and specifically, CRUs perform distributed beamforming to DUs. This scenario is suitable for IoT applications as in [44], and in the multiple user cooperative network, the need for using the NOMA with beamforming technique is imperative for IoT applications, while the beamforming technique can be employed as in [45]. In the second case study, energy-efficient NOMA along with energy-harvesting techniques are analyzed, to understand the feasibility of NOMA as an energy-efficient transmission protocol for a multiple access cooperative network. Finally, in our third case study, we include a special case, where the SC-FDMA technique is used in a dynamic point-to-point communication with varying channel conditions, which requires a robust channel estimation technique along with a CFO estimation technique to estimate the channel condition. Once again, the focus is on energy efficiency, in this case from the communications point of view, including a receiver design with joint detection and channel estimation.

All three case studies are focused on improving the energy efficiency of the 5G network. Case Study 1 and Case Study 2 illustrate energy-harvesting techniques to enable user cooperation and improve the overall performance of the system, whereas Case Study 3 illustrates the idea of accommodating the energy-harvesting technique along with the CFO and channel estimation techniques. The results of the case studies contribute towards the energy efficiency of 5G networks while enhancing the data rate performances.

## 3. Case Study 1: Wireless Power Transfer-Enabled Data Rate Fairness Beamforming

**Figure 1:**
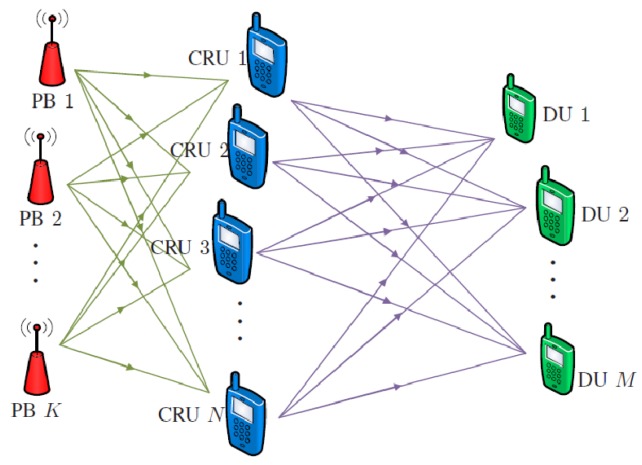
System model. PB, power beacon; CRU, cooperative relay user; DU, destination user.

We consider a cooperative communication system with *N* CRUs, *M* DUs, and *L* PBs, as shown in Figure 1. We assume all the CRUs, DUs, and PBs are equipped with a single antenna capable of performing energy harvesting and information transfer in a time-switching manner. The link between the nodes experience independent frequency flat Rayleigh fading with the free space path loss model. The communication happens in two phases, namely energy harvesting and information transmission.

During the energy-harvesting phase, the PBs perform beamforming to maximize the total power delivered to all the CRUs as:

(1) $$\begin{array}{cc} \max_{z} & \sum_{i=1}^{N}\frac{\eta_{R_{i}}T_{P}}{T_{I}}z^{†}H_{R_{i}}z\\ \mathrm{subject}\;\mathrm{to} & ∥z_{k}{∥}_{2}^{2}\leq P_{P_{k}},\;\;k=1,\cdots ,L, \end{array}$$

where z is the beamforming vector, $H_{R_{i}}$ is the channel matrix to the *i*th CRU, $\eta_{R_{i}}$ is the energy-harvesting efficiency of the *i*th CRU, $T_{P}$ is the energy harvesting-time slot, $T_{I}$ is the information transmission time slot, and $P_{P_{k}}$ is the power constraint at the *k*th PB.

Then, during the information transmission phase, the CRUs cooperatively send the beamforming different information to the DUs simultaneously. In such a system, the best data rate with fairness can be achieved by maximizing the minimum of the data rate at the DUs, which is equivalent to maximizing the minimum of the signal-to-interference plus noise ratio at the DUs with respect to the available transmit power at each CRU [46], which can be expressed mathematically as:

(2) $$\begin{array}{cc} \max_{g_{D_{1}},\cdots ,g_{D_{M}}}\underset{j=1,\cdots ,M}{\;\min} & \frac{g_{D_{j}}^{†}H_{D_{j}}g_{D_{j}}}{\sum_{\begin{array}{c} k=1\\ k\neq j \end{array}}^{M}g_{D_{k}}^{†}H_{D_{j}}g_{D_{k}}+\sigma_{D_{j}}^{2}}\\ \mathrm{subject}\;\mathrm{to} & ∥g_{R_{i}}{∥}_{2}^{2}\leq P_{R_{i}},\;\;i=1,\cdots ,N, \end{array}$$

where $g_{D_{j}}$, $H_{D_{j}}$, and $\sigma_{D_{j}}^{2}$ are the beamforming coefficient, channel matrix, and additive white Gaussian noise at the *j*th DU and $g_{R_{i}}$ and $P_{R_{i}}$ are the beamforming coefficients and harvested power constant at the *i*th CRU, respectively.

Then, the system performance can be further improved by jointly optimizing the energy beamforming and information beamforming coefficients. According to the open literature, the joint energy beamforming and data rate fairness beamforming problems have not been addressed before. The joint optimization problem can be expressed as:

(3) $$\begin{array}{cc} \max_{g_{D_{1}},\cdots ,g_{D_{M}}}\underset{j=1,\cdots ,M}{\;\min} & \frac{g_{D_{j}}^{†}H_{D_{j}}g_{D_{j}}}{\sum_{\begin{array}{c} k=1\\ k\neq j \end{array}}^{M}g_{D_{k}}^{†}H_{D_{j}}g_{D_{k}}+\sigma_{D_{j}}^{2}}\\ \mathrm{subject}\;\mathrm{to} & ∥g_{R_{i}}{∥}_{2}^{2}\leq \frac{\eta_{R_{i}}T_{P}}{T_{I}}z^{†}H_{R_{i}}z,\;\;i=1,\cdots ,N,\\ & {\left|z\left(k,1\right)\right|}^{2}\leq P_{P_{k}},\;\;k=1,\cdots ,L. \end{array}$$

The problem in (1) is quadratic and can be transformed into a convex problem and solved using semidefinite relaxation. Then, the problem in (2) and the problem in (3) are not tractable and non-convex. However, these problems can be reformulated into a quasi-convex problem by introducing and auxiliary variable and solved using the semidefinite relaxation technique and the bisectional method [46].

### Numerical Results

**Figure 2:**
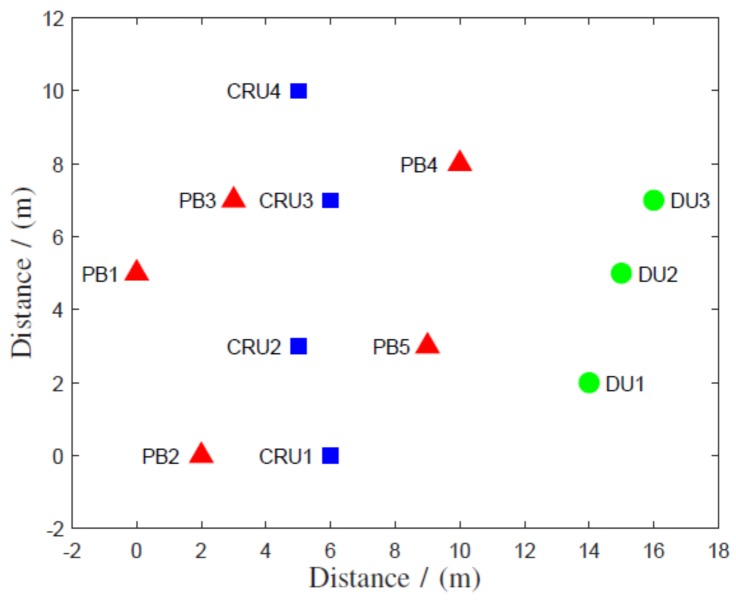
System coordinates.

In this section, we consider the above system in Figure 1 with four CRUs, three DUs, and five PBs to show the performance. The coordinates of nodes were mapped as shown in Figure 2. Further, we assumed the information and energy symbol duration were $1\times 10^{-6}$ s. The energy-harvesting efficiency of all CRUs was 60%. The channel gains were assumed to be independent frequency flat Rayleigh distributed. The free space path loss model was considered with a path loss exponent of 4, $d_{0}=1$ m, and 1 GHz of operating frequency. It was assumed that the additive white Gaussian noise power spectral density was −173.83 dBm/Hz and that the total bandwidth was 5 MHz.

In the above system, independent energy harvesting (I EH) and data rate fairness beamforming (DRF BF) with distributed PBs (DRF BF) were developed by solving the energy beamforming problem in (1) and the data rate fairness beamforming problem in (2) in the respective time slots. Then, joint energy harvesting and data rate fairness beamforming with distributed PBs (J EH and DRF BF with DPBs) was developed by solving the joint optimization problem in (3).

Then, we compared the above system with a similar system with single multiple antenna PB (SMAPB). In this system, we considered independent energy harvesting and data rate fairness beamforming (I EH and DRF BF with SMAPB) and joint energy harvesting and data rate fairness beamforming (J EH and DRF BF with SMAPB).

**Figure 3:**
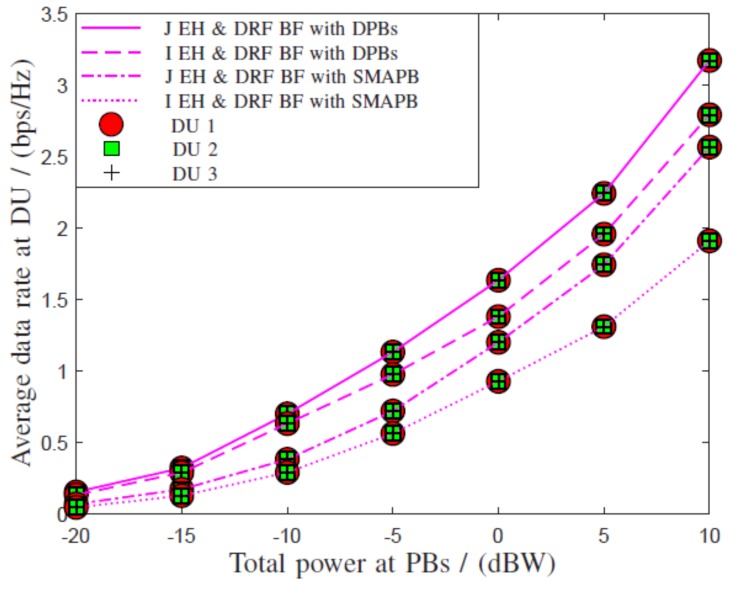
Comparison of data rates at DUs for the considered systems and beamforming techniques with the total power available at PBs. EH, energy harvesting; SMAPB, single multiple antenna PB.

Figure 3 shows that all the above beamforming schemes ensure equal data rates to DUs and provide data rate fairness. Then, the system with distributed PBs outperformed the system with SMAPB regardless of independent or joint beamforming. Furthermore, joint beamforming outperformed the corresponding independent beamforming for both systems. Therefore, the proposed J EH and DRF BF with DPBs is the ideal choice for the overall performance improvement of such system.

## 4. Case Study 2: Built-In Energy-Efficient Modulation-Based NOMA

### 4.1. Proposals for SWIPT-Enabled M-NOMA

Many scientists are working toward the perfect implementation of NOMA as one of the 5G technologies. In the simplest NOMA techniques, we added the signals intended for different users (say, users are near or far from the transmitter), which were separate in power (in this sense, the transmitted signals resembled the ones associated with the hierarchical constellations used for broadcasting channels [47]). The modulation-based NOMA (M-NOMA) scheme is one of the efficient techniques to improve conventional NOMA. M-NOMA provides better efficiency, data rate, symbol error rate, less system complexity, and minimizes interference [48]. In M-NOMA, a regular M-QAM modulation is used for the signal transmission, similarly to conventional NOMA. Even though both schemes use regular modulations, M-NOMA differs from the conventional NOMA by allocating the real and the imaginary part of the constellation points for different users to avoid signal interference, and the user decodes either the real or imaginary part of the signal based on the allocation [48].

In the downlink, the conventional NOMA base station (BS/transmitter) broadcasts signals of all users after superposing with suitable power levels (a far user requires more power than a near one due to transmission loss) and modulates (each modulation technique has a real and imaginary component). Users, which are in the vicinity of BS, receive the superposed signal. Each near user decodes the signal of all other users, then subtracts it from the total received signal. This process is called successive interference cancellation (SIC). Each far user decodes/downloads its own signal with the consideration of other signals as interference.

In M-NOMA, BS modulates near users on the real component of the QPSK modulation and far users on the imaginary component of the QPSK modulation. It creates orthogonality between near and far users, which ideally reduces the interference between near and far users. Therefore, near users do not have to apply SIC for far users’ signals. The near user only applies SIC for the near users’ signals. NOMA increases the complexity of the system due to a greater number of SICs on the receiving end of near users due to the high power signals of far users. However, M-NOMA requires SIC to be 0.5-times, less SIC than NOMA, due to the orthogonality created during modulation.

In cooperative NOMA, the near user decodes the signal of the far user and assists the far user by relaying the signal. Hence, the far user receives the signal from the BS and near user. It then combines both signals and obtains better QoS. To make the system energy efficient, the near user harvests energy from the received signal from the BS and uses the harvested energy for transmission of far users’ signals, which saves the energy of the near user. However, the near user can use this energy for any other purpose, as well. In M-NOMA, due to the orthogonality created between near and far users during modulation, each user can harvest more energy than NOMA. For example, through power splitting, near U1 can decode its signal and harvest energy from its own signal and U2’s signal. Additionally, U1 can also harvest energy from U3’s and U4’s signals without splitting power as U1 receives their signals and does not decode their signals. Therefore, in M-NOMA, each user can harvest more energy than NOMA. Table 1 briefly explains the difference between NOMA and M-NOMA.

### 4.2. M-NOMA Communication

**Figure 4:**
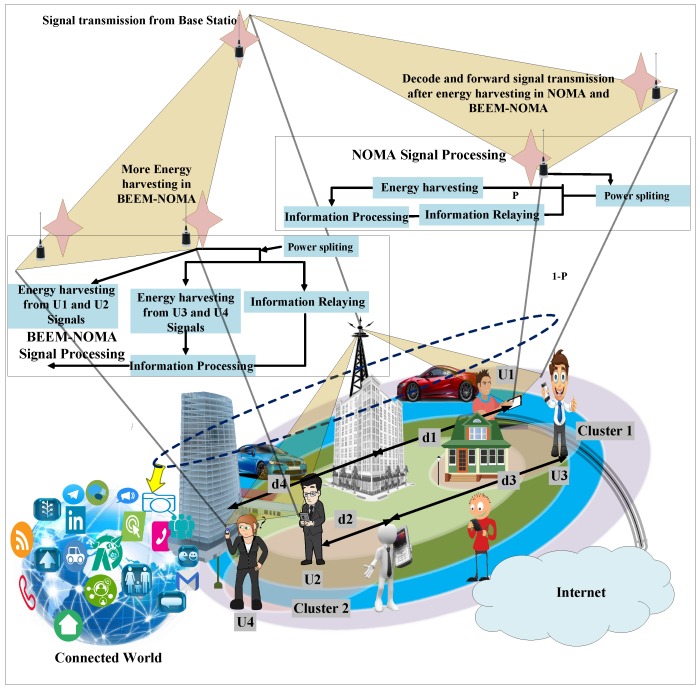
Schematics of the PTP and cooperative SWIPT-enabled M-NOMA and NOMA scenario.

In the given four-user system of Figure 4, we have analyzed the DL NOMA and M-NOMA for one BS, Rayleigh flat fading transmission channel, and path loss coefficient is chosen according to distance, power coefficient $\alpha =\alpha_{1}+\alpha_{2}+\alpha_{3}+\alpha_{4}=1$, total power $P_{T}$, and QPSK modulation. In both NOMA and M-NOMA point to point (PTP) communication systems, all users harvest energy from the signal received from the BS. Each user is capable of harvesting energy from the received superposed signal. Table 1 lists the difference of harvested energy for each individual user. It should be noted that for a fair comparison, the amount of transmitted power and other physical components for all users are the same for NOMA and M-NOMA. Table 1 lists the difference of harvested energy for each individual user. It shows that each NOMA user can harvest energy with power splitting, as shown in Figure 4. However, in M-NOMA, each user can use the power splitting ratio for signal processing of its own components (real or imaginary) and the received power of the other constellations’ signals without splitting its power. Hence, M-NOMA is a built-in energy-efficient scheme (BEEM-NOMA).

We use the following formulae for harvesting energy from the received signal:

(4) $$\zeta_{h}=\frac{\eta_{eh}P_{T}\left|g\right|\alpha T}{d^{m}}.$$

where $\zeta_{h}$ is the harvested energy, $\eta_{eh}$ is the efficiency of harvested energy, |g| is the channel power gain, α is the power splitting factor, *T* is the time, *d* is the distance, and *m* is the path loss factor.

For cooperative communications, we chose two clusters. U1 and U2 are in Cluster 1, where U1 processes the signal decode-and-forward and harvested energy for U2. Similarly, U3 relays information to U4 after harvesting energy, as shown in Figure 4.

### 4.3. M-NOMA: An Efficient System

M-NOMA is an extended technique of NOMA. The difference between NOMA and M-NOMA begins from the superposition of the signal by the BS before transmission. In M-NOMA, we consider the four-user system of M-NOMA as shown in Figure 4. The BS modulates near and far users on separate modulation components. In this paper, the BS modulates near users’ (U1 and U2) signals on the real component and far users’ (U3 and U4) signals on the imaginary component of the QPSK modulation technique. The BS broadcasts the superposed signal after modulation. When U1 receives the signal, it applies SIC for U2 only. With prior knowledge, it decodes the signal from the real component of the signal. In the case of cooperative communication, it decodes the signal, harvests energy (when needed), and then forwards U2’s signal to assist U2. Similarly, U3 decodes its signal from the imaginary component. In case of N users, the number of SICs is N/2.

**Figure 5:**
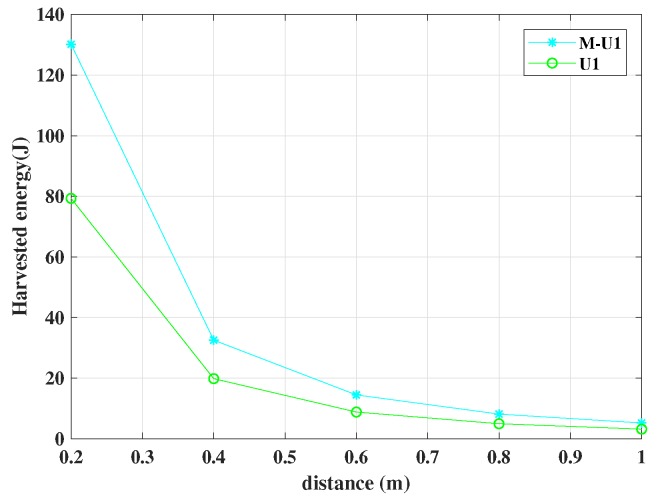
Harvested energy vs. distance for BEEM-NOMA (M-U1) and NOMA (U1) with energy efficiency $\eta_{ee}=60\%$. BEEM-NOMA outperforms NOMA.

We used MATLAB to simulate EH. Figure 5 shows the simulation results for harvested energy with respect to the distance of U1 from the BS for NOMA and BEEM-NOMA. We considered η=60%, $P_{T}=1$ W. It can be seen from the figure that the amount of energy harvested decreased with the distance. However, BEEM-NOMA outperformed NOMA.

**Figure 6:**
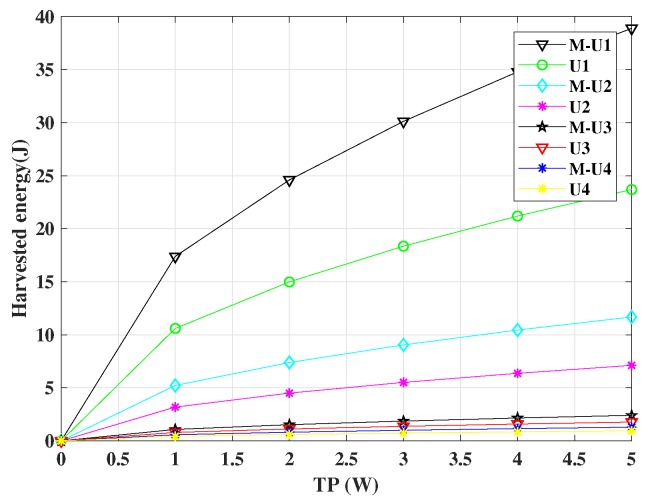
Harvested energy vs. transmit power for BEEM-NOMA (M-U1, M-U2, M-U3, and M-U4) and NOMA (U1, U2, U3, and U4).

Figure 6 shows the simulation result of harvested energy vs. $P_{T}$. In this simulation, we considered η=60%, $P_{T}=1$–5 W, power allocation coefficient α=0.085+0.157+0.337+0.421=1, and T = 1 s. Since the time is 1 s, the harvested energy can be considered as harvested power. The simulation results show that BEEM-NOMA users harvested more energy than each comparative user, since BEEM-NOMA has built-in energy efficiency.

## 5. Case Study 3: Receiver Designing to Employ SWIPT with Joint CFO and Channel Estimation

### 5.1. System Model

**Figure 7:**
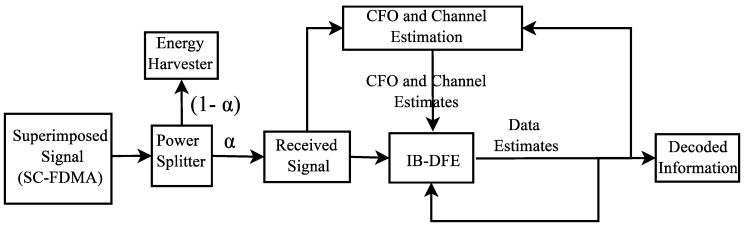
Block diagram of the receiver for SWIPT with joint carrier frequency offset (CFO) and channel estimation. SC-FDMA, single-carrier frequency-division multiple access; IB-DFE, iterative block decision feedback equalization.

The system used quadrature phase shift keying (QPSK) modulation with SC-FDMA over Rayleigh fast fading channel ${}^{\prime \prime }H_{l}^{\prime \prime }$. It is assumed that the system experiences additive white Gaussian noise (AWGN) with variance $N_{0}/2$ modeled as a zero mean complex Gaussian random variable and undergoing phase rotation. The system adopted power splitting protocol-based SWIPT (PS-SWIPT), and the receiver split the received signal using a special circuit on the basis of the power allocation ratio [4]. The power allocation ratio for the information decoding (ID) and EH was α and (1−α), respectively, where 0<α<1. The receiver estimated the CFO, the channel, and information using the iterative receiver, as illustrated in Figure 7.

The frame structure of the signal was similar to the frame structure used in [49], where the pilot and the information signals were superimposed together as a single signal. The pilot and the information signals are denoted as $X_{k,l}^{\left(\Delta f\right)}$ and $Q_{k,l}^{\left(\Delta f\right)}$, respectively, and the CFO of the signal is denoted as Δf. The transmit powers of $X_{k,l}^{\left(\Delta f\right)}$ and $Q_{k,l}^{\left(\Delta f\right)}$ are denoted as $P_{x}$ and $P_{q}$, respectively. In the frame, the time duration per symbol and block duration are denoted as *T* and $T_{l}$, respectively. The frame structure of the signal had *L* number of signal blocks with each signal blocks having *K* number of symbols. The energy harvested at the receiver is denoted as $E_{y}$ and is written as:

$$\begin{array}{c} E_{y}=\eta_{eh}\left(1-\alpha \right)\left(P_{x}+P_{q}\right)h_{l}T_{l}, \end{array}$$

where $\eta_{eh}$ is the EH efficiency of the rectenna and $h_{l}$ is $|H_{l}{|}^{2}$. $|H_{l}{|}^{2}$ is the channel power gain of $H_{l}$ with $|H_{l}{|}^{2}∼e^{\sigma_{SD}^{2}}$ and $\sigma_{SD}^{2}={d_{SD}}^{-\chi }$, where $e^{\sigma_{SD}^{2}}$ is the exponential distribution mean of $H_{l}$. The distance between *S* and *D* is denoted as $d_{SD}$, and the path loss factor is denoted as χ. The superimposed signal for the *l*th block of the received signal with CFO is denoted as $Y_{k,l,i}^{\left(\Delta f\right)}$. $Y_{k,l,i}^{\left(\Delta f\right)}$ can be written as:

$$\begin{array}{c} Y_{k,l,i}^{\left(\Delta f\right)}=\alpha \left(H_{k,l}\left(\sqrt{P_{x}}X_{k,l}^{\left(\Delta f\right)}+\sqrt{P_{q}}Q_{k,l}^{\left(\Delta f\right)}\right)+W_{l}\right)\\ \;+W_{e,l}, \end{array}$$

where *k* is the frequency of block *l*, k=0,1,…,K−1 and l=0,1,…,L−1. $W_{l}$ and $W_{e,l}$ are the AWGN created due to the signal transmission and the power splitting operation, respectively. To decode information from $Y_{k,l,i}^{\left(\Delta f\right)}$, the receiver follows three steps:

- Estimate CFO of $Y_{k,l,i}^{\left(\Delta f\right)}$ by using the Moose technique [29]. The mean CFO estimate is more accurate with the increase in the number of signal blocks. Compensate the CFO of $Y_{k,l,i}^{\left(\Delta f\right)}$ with the mean CFO estimate value.
- Compute the average channel estimate over *l* blocks, and compute the information estimate using the average channel estimate.
- To improve the accuracy of the decoding process, the IB-DFE receiver was used to improve the information estimates, and again, the information estimates were recursively used to improve the channel estimate in a feedback loop, as in [37].

The information estimate and channel estimate that were obtained from the iterative receiver are denoted as ${\tilde{X}}_{k,l}^{\left(j,\Delta f\right)}$ and ${\tilde{H}}_{k,l}^{\left(j\right)}$, respectively, where *j* is the number of iterations followed in the IB-DFE receiver and j=0,1,…,J. The final information estimate after the optimum number iteration is written as:

$$\begin{array}{c} {\tilde{X}}_{k,l}^{\left(j,\Delta f\right)}=\left(Y_{k,l,i}^{\left(\Delta f\right)}-\alpha \sqrt{P_{q}}Q_{k,l}^{\left(\Delta f\right)}{\tilde{H}}_{k,l}^{\left(j\right)}\right)F_{k,l}^{\left(j\right)}\\ -{\tilde{X}}_{k,l}^{\left(j-1,\Delta f\right)}B_{k,l}^{\left(j\right)}, \end{array}$$

where ${\tilde{X}}_{k,l}^{\left(j-1,\Delta f\right)}$ is the previous iteration value of ${\tilde{X}}_{k,l}^{\left(j,\Delta f\right)}$. In the first iteration, the average channel estimate was used to estimate information in the IB-DFE receiver. $F_{k,l}^{\left(j\right)}$ and $B_{k,l}^{\left(j\right)}$ are the feed-forward and feedback coefficients, respectively, and they were computed as in [34].

The CFO and channel estimation technique in this model were principally based on the superimposed signal as implemented in [34,37]. The error rate performance of this technique can be further improved by optimizing the ratio between $P_{x}$ and $P_{q}$ based on the power of the pilot signal, information signal, and noise power at the receiver, as mentioned in [37]. The power of the pilot signal, information signal, and noise power at the receiver are denoted as $\rho_{q},\rho_{x}$, and $\rho_{w}$, respectively. The relation between $\rho_{q}$ and $\rho_{x}$ and $\rho_{x}$ and $\rho_{w}$ is given as $\beta_{Q}=\frac{\rho_{q}}{\rho_{x}}$ and $\beta_{X}=\frac{\rho_{x}}{\rho_{w}}$, respectively.

### 5.2. Numerical Results

In this section, we illustrate the simulation results of the system model from [37] for analysis purposes. We set N=256, $T_{l}=1$ second, and L=3, and assumed $\eta_{EH}=0.9$, χ=2, Δf=0.1, $d_{SD}=3$ m, α=0.7.

**Figure 8:**
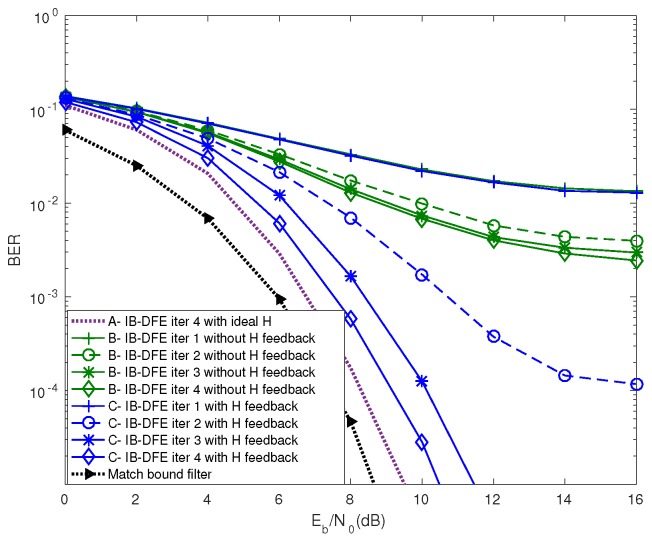
Comparison of the BER performance of the system based on two different methods to estimate information.

Figure 8 demonstrates the BER performance of the system based on two different methods to estimate information using the iterative receiver, where $P_{x}=25$ dBm and $P_{q}=21$ dBm. The curve *A*, *B*, *C*, and *D* denotes the information estimate with the ideal channel condition, with IB-DFE receiver feedback for the information estimate, with IB-DFE receiver for information and channel estimates, and with the match bound filter, respectively. Figure 8 demonstrates the improved BER performance of *C* as compared to *B*, because *C* uses both the channel estimate feedback and information estimate feedback recursively in the IB-DFE receiver. The BER performance of *A* and *D* is plotted to compare and validate our estimation techniques.

The results of $P_{si}$, $E_{y}$, and $E[{\varepsilon_{X}}_{k,l,F}^{\left(j,\Delta f\right)}]$ are based on the value of $P_{q}$. $P_{x}=25$ dBm, and $P_{q}$ varied from 14 dBm–21 dBm, as illustrated in Table 2. With the increase in $P_{si}$, $E_{y}$ increased proportionally. The value $E[{\varepsilon_{X}}_{k,l,F}^{\left(j,\Delta f\right)}]$ decreased with the increase in $P_{q}$, up to a certain limit.

**Figure 9:**
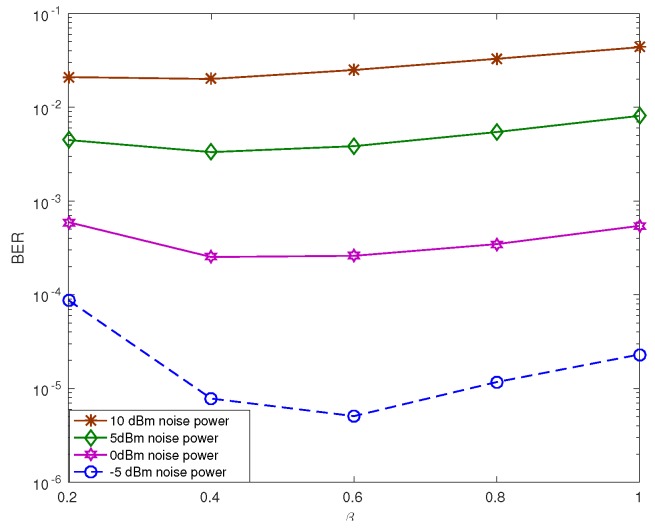
BER performance based on the ratio of the power between the pilot signal and the information in the superimposed signal.

Figure 9 demonstrates the BER performance based on $\beta_{Q}$ at the receiver, where χ=2, $d_{SD}=3$ m, α=0.3, $P_{si}=28$ dBm, Δf=0, and L=1. The value of $\rho_{w}$ was fixed at −5 dBm, 0 dBm, and 5 dBm, respectively. For $\rho_{w}=5$ dBm and $\rho_{w}=-5$ dBm, the optimum $\beta_{Q}$ value was 0.4 and 0.6, respectively. The optimum value of $\beta_{Q}$ changes depending on the SNR condition at the receiver. The optimum $\beta_{Q}$ value depicts that the power allocated for the pilot signal increases with the increase in SNR, i.e., $\beta_{X}$, to improve the channel estimation performance as much as possible by taking advantage of the high SNR condition.

## 6. Conclusions

In this paper, we first considered the integration of distributed energy beamforming with a cooperative communication system consisting of multiple cooperative relays and users, where multiple cooperative relays performed the data rate fairness beamforming to maintain fair service to all users. In contrast to the traditional cooperative communication systems, relays harvested the energy for cooperation from energy beamforming. We have studied several energy harvesting schemes for the cooperative relay and user system, and then, finally, we demonstrated that the joint energy harvesting and data rate fairness beamforming with distributed power beacons were the best-performing schemes.

Furthermore, for the utilization of available natural resources like the radio spectrum and energy, we have proposed an enhanced version of SWIPT-enabled M-NOMA, BEEM-NOMA. It has been observed that BEEM-NOMA offered an improved system efficiency in terms of harvested energy; here, for half of the users, harvested energy without additional power consumption. Finally, in the third case study, we demonstrated an SWIPT model that harvests energy from the superimposed signal, which is used for estimating carrier frequency offset and channel condition. The channel and information estimates, respectively, were improved by employing the iterative block decision feedback equalization at the receiver. The optimum transmit power ratio between the pilot and information signal was calculated for the best error rate performance of the system under varying SNR conditions.

## Figures and Tables

**Table 1:** Assessment of the individual energy-harvesting capability of NOMA and BEEM-NOMA. SIC, successive interference cancellation.

System Evaluation	Receiver	U1	U2	U3	U4
**Decode**	NOMA	U1, U2, U3 and U4	U2, U3 and U4	U3 and U4	U4
	M-NOMA	U1 and U2	U2	U3 and U4	U4
**SIC**	NOMA	U2, U3, and U4	U3 and U4	U4	N/A
	M-NOMA	U2	N/A	U4	N/A
**Interference cancellation**	NOMA	No	No	No	With U1, U2, and U3
	M-NOMA	No	No	No	With U3 only
**Energy-harvested signals**	NOMA	Power splitting of U1’s, U2’s, U3’s, and U4’s signals	Power splitting of U1’s, U2’s, U3’s, and U4’s signals	Power splitting of U1’s, U2’s, U3’s, and U4’s signals	Power splitting of U1’s, U2’s, U3’s, and U4’s signals
	M-NOMA	Power splinting of U1’s and U2’s signals and directly without power splitting from U3’s and U4’s signals	Power splitting of U1’s and U2’s signals and directly without power splitting from U3’s and U4’s signals	Power splitting of U3’s and U4’s signals and directly without power splitting from U1’s and U2’s signals	Power splitting of U3’s and U4’s signals and directly without power splitting from U1’s and U2’s signals

**Table 2:** The amount of energy harvested at the receiver and the expected value of the information estimate error based on the power of the pilot signal.

$P_{q}$ (dBm)	15	16	17	18	19	20	21
$P_{si}$ (dBm)	25.4139	25.5150	25.6389	25.7901	25.9732	26.1933	26.4554
EH (mJ)	0.0216	0.0221	0.0227	0.0235	0.0245	0.0258	0.0274
$E[{\varepsilon_{X}}_{k,l,F}^{\left(j,\Delta f\right)}]$ at 5 dB SNR	0.2756	0.2105	0.1815	0.1594	0.1559	0.1525	0.1411
$E[{\varepsilon_{X}}_{k,l,F}^{\left(j,\Delta f\right)}]$ at 10 dB SNR	0.0176	0.0143	0.0138	0.0138	0.0137	0.0138	0.0137
$E[{\varepsilon_{X}}_{k,l,F}^{\left(j,\Delta f\right)}]$ at 15 dB SNR	0.0136	0.0135	0.0135	0.0135	0.0135	0.0135	0.0135
